# Radical cystectomy for bladder cancer: A single center experience

**DOI:** 10.4103/0970-1591.38604

**Published:** 2008

**Authors:** Narmada P. Gupta, Surendra B. Kolla, Amlesh Seth, Prem N. Dogra, Ashok K. Hemal, Rajeev Kumar, Sabyasachi Panda

**Affiliations:** Department of Urology, All India Institute of Medical Sciences, New Delhi, India

**Keywords:** Bladder cancer, pelvic lymphadenectomy, radical cystectomy

## Abstract

We present the outcomes of a large series of patients treated with radical cystectomy and pelvic lymphadenectomy for transitional cell carcinoma of bladder.

**Materials and Methods:**

A total of 502 patients underwent radical cystectomy (RC) for bladder cancer from 1992 till December 2006. Of these, 432 patients with primary transitional cell carcinoma of bladder underwent RC with bilateral pelvic lymphadenectomy with a curative intent. The clinical course, pathologic characteristics and long-term clinical outcomes were evaluated in this group of patients.

**Results:**

The median follow-up was 62 months. There were 30 (6.9%) perioperative deaths and 111(25.7%) early complications. The recurrence-free survival (RFS) and overall survival (OAS) were 66% and 62% at five years and 62% and 40% respectively at 10 years. The RFS and OAS were significantly related to the pathological stage and lymph node status with increasing pathological stage and lymph node positivity associated with higher rate of recurrence and worse OAS *(P < 0.001)*. A total of 145 patients (33.5%) developed bladder cancer recurrence. Of these, 40 (27.6%) developed local pelvic recurrence and 105 patients (72.4%) developed distant recurrence. The median time to local and distant recurrence was 12 and 16 months respectively.

**Conclusion:**

The clinical results reported from this large group of patients demonstrate that radical cystectomy provides good survival results for invasive bladder cancer patients with low incidence of pelvic recurrence.

## INTRODUCTION

Radical cystectomy (RC) and bilateral pelvic lymphadenectomy (B/L PLND) is considered the treatment of choice for treatment of patients with muscle invasive and refractory high-grade superficial bladder cancer. Several large series have shown excellent five and 10-year survival rates after RC, particularly for patients with organ-confined node negative disease.[1–3] With increasing experience, a considerable decrease in the mortality and morbidity has been noticed. Radical cystectomy provides accurate staging information, which is the key for decision regarding adjuvant treatment options, while staging by clinical means is associated with up to 30% staging error.[3] Several advancements in the recent past have contributed to decreasing the morbidity, improving survival and quality of life following RC which include: laparoscopic and robotic-assisted radical cystectomy,[4,5] extended lymphadenectomy templates,[6] increased use of continent urinary diversion,[7] nerve-sparing RC,[8] neo-adjuvant and adjuvant chemotherapy.[9]

Our hospital is a tertiary referral center and is one of the few high-volume centers treating bladder cancer patients in the country.[10] In the present study we have reviewed our long-term surgical experience and clinical outcomes in a large group of patients treated with RC and B/L PLND for muscle-invasive bladder cancer or superficial cancer refractory to intravesical therapy over the past 15 years.

## MATERIALS AND METHODS

### Patient population

A total of 502 patients underwent RC for bladder cancer from 1992 till December 2006; out of which 41 had non-transitional cell (TCC) bladder cancer, nine underwent salvage cystectomy for invasive recurrence after chemo radiotherapy, seven had other pelvic malignancies, seven had metastases at the time of cystectomy and six had inoperable bladder cancer identified at the time of laparotomy. The remaining 432 patients with primary TCC of bladder underwent RC+PLND with a curative intent and are the focus of this analysis. The patients' clinical characteristics are shown in Table 1. The indication for cystectomy was tumor either ≥ T2 stage or superficial tumor refractory to transurethral resection and intravesical chemotherapy and/or immunotherapy. Ninety per cent of patients demonstrated high-grade bladder tumors.

**Table 1:** Clinical and pathological characteristics of patients who underwent RC+B/L PLND

Characteristic	No. pts (%)
Age (years; range)	62.3 (30-85)
Sex	
Male	362(83.8)
Female	70(16.2)
Pathological staging	
pT1	26(6)
pT2	160(37)
pT3a/3T3b	90/104(20.9/24.1)
pT4	52(12)
N0	324(75)
N+	108(25)
Grade	
Low	45(10.4)
High	387(89.6)
Histology	
Pure transitional cell carcinoma	454(91.7)
Pure squamous cell carcinoma	13(2.6)
Pure adenocarcinoma	18(3.6)
Small cell cancer	10(2.1)
Approach to cystectomy	
Open	372(86.1)
Laparoscopic	48(11.1)
Robotic	12(2.8)
Adjuvant chemotherapy	
Yes	125(29)
No	307(71)
Urinary diversion	
Ileal conduit	346(80.1)
Orthotopic neobladder	47(10.9)
Rectosigmoid pouch	26(6)
Cutaneous ureterostomy	9(2.1)
Continent cutaneous	4(0.9)

A total of 60 patients had obstructive uropathy at presentation with a mean serum creatinine of 9 mg% (range 2.4-16.5 mg%). Initial management of uremia in these patients was based on various clinical and laboratory findings, such as 1) Scr at presentation, 2) general patient condition and performance status, 3) evidence of sepsis and 4) disease stage. Of these, 40 patients (9.2%) underwent radical cystectomy after their renal function was stabilized with percutaneous nephrostomy (PCN) ± hemodialysis.

### Treatment

Neoadjuvant chemotherapy or radiotherapy was not used in any patient. All patients underwent B/L PLND, RC and urinary diversion. Pelvic lymphadenectomy was performed either before cystectomy, en bloc or after cystectomy depending on the tumor bulk and configuration of the pelvis. Urinary diversion was either incontinent or continent [Table 1]. In patients who underwent laparoscopic or robotic RC, urinary diversion was done extracorporeally through the specimen extraction incision. Adjuvant chemotherapy was used in patients with pathological stage ≥ T3b and/or lymph node involvement (29%). Regimens consisted of methotrexate (M), vinblastine (V), doxorubicin (A) and cisplatin (C) in 21%, CMV in 47%, gemcitabine and cisplatin in 27%, other cisplatin or carboplatin-based regimens in 5%. Adjuvant therapy was administered at the discretion of the treating physician.

### Pathology

The 1997 TNM classification was used for pathological staging and 1973 WHO classification was used for pathological grading. Multiple sections were obtained from the tumor, bladder wall, mucosa adjacent and distant from tumor along with ureters and lymph nodes. In men, sections from seminal vesicles and prostate were obtained while in women, sections were obtained from ovaries, uterus and vagina when appropriate.

### Follow-up

The patients were seen at three-monthly intervals during the first two years, six-monthly in the third year and yearly thereafter. Follow-up comprised history with physical examination, biochemical profile, chest radiography and ultrasonography of the abdomen. Based on this preliminary evaluation, further imaging by abdominal/pelvic computerized tomography or bone scan were performed for suspected local or distant recurrences. Follow-up was available for 360/432 (83%) patients. The mean and median follow-up for the entire cohort of 432 patients was 66 (8-120 months) and 62 months. Seventy per cent of our patients had ≥ three years follow-up and 52% had ≥ five years follow-up.

### Clinical outcome

Perioperative mortality (death within 30 days of surgery or before discharge), early (within three months of surgery) and late complications were noted. Outcomes were measured as time to clinical recurrence or overall survival (OAS). Recurrence-free survival (RFS) was calculated as the time from cystectomy to the date of first documented clinical recurrence or until last follow-up if the patient had not developed recurrence. Survival was calculated as the time from cystectomy to the date of death. All deaths were counted as events; patients who are still alive were censored at the date of last contact. Recurrences were classified as local (within the field of surgery) or distant or both.

### Statistical analysis

Kaplan-Meier plots were used to estimate OAS and RFS for all the patients combined and for subgroups classified by pathological stage. Survival rates were evaluated using Kaplan-Meier method and logrank test. Multivariate analysis was performed using Cox-proportional hazard method. *P* value < 0.05 was considered significant. All *P* values were two-sides. All analysis was performed with SPSS^®^, version 13.0.

## RESULTS

### Mortality

Perioperative death occurred in 30 (6.9%) of 432 patients. The causes of perioperative mortality are shown in Table 2. There was no obvious difference in mortality rate with respect to urinary diversion employed: 25 perioperative deaths among 355 (7%) patients with non-continent diversion and 5/77 (6.5%) patients with continent diversion.

**Table 2:** Perioperative mortality from radical cystectomy

Category	No. periop mortality	Median patient age at surgery (range)	Median days to death (range)
Cardiovascular	6	62 (55-65)	6(2-20)
Acute myocardial	4		
infarction	2		
Arrhythmia		
Infectious/sepsis	16	60(45-76)	18(12-31)
Urine leak	2		
Bowel leak/fistula	4		
Chest infection	10		
ARDS	2	58(55-60)	21(17-25)
Pulmonary embolus	3	68(63-72)	3(0-7)
Hemorrhage	1	57	1
Unknown	2	65(60-70)	25(20-30)

### Morbidity

A total of 111 patients (25.7%) developed early complications. Of these, 27 patients (6.25%) developed diversion-related and 84 (19.4%) developed diversion unrelated complications. Forty-three patients (10%) developed late complications. The various complications encountered in our patients are shown in Table 3. There was no obvious difference observed in the complication rate with respect to the urinary diversion employed: 93/355 (26.2%) in non-continent diversion and 18/77 (23.4%) in the continent diversion group.

**Table 3:** Postoperative complications after radical cystectomy

Early complication	N
Diversion-related	27
Bowel anastomotic leak	8
Urine leak (no intervention)	12
Urine leak (intervened)	7
Diversion unrelated	84
Deep vein thrombosis	10
Small bowel obstruction	18
Wound dehiscence	12
Pneumonia	11
Myocardial infarction	6
Disseminated intravascular coagulation	2
Renal failure	5
Acute respiratory distress syndrome	4
Pyelonephritis	16
Late complications	43
Intestinal obstruction	18
Incisional hernia	10
Parastomal hernia	1
Stomal stenosis	1
Neobladder perforation	2
Urolithiasis	2
Metabolic acidosis	3
Uretero-ileal anastomotic stricture	6

### Pathological staging

Pathological substaging of our patients is shown in Table 1. Pathological subgroups include 175 patients (40.5%) with organ-confined node-negative tumors, 149 (34.5%) with extravesical node-negative tumors and 108 (25%) with lymphnodal involvement. The incidence of lymphnodal involvement increased with increasing pathological stage of primary tumor: 1/26 (3.8%) with pT1, 23/160 (14.4%) with pT2, 21/90 (23.3%) with pT3a, 39/104 (37.5%) with pT3b and 24/52 (46.1%) with pT4 tumors.

### Survival

**Figure 1:**
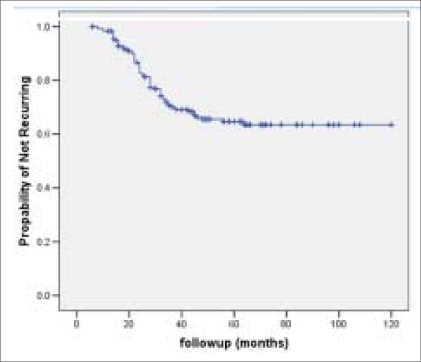
Kaplan-Meier cumulative recurrence-free survival curve

**Figure 2:**
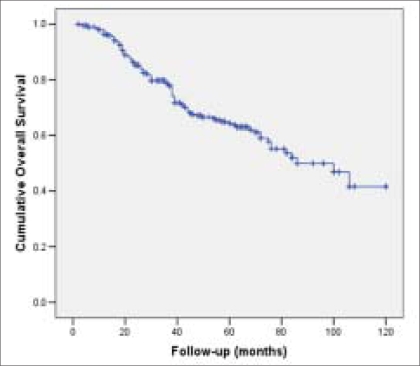
Kaplan-Meier cumulative overall survival curve

**Figure 3:**
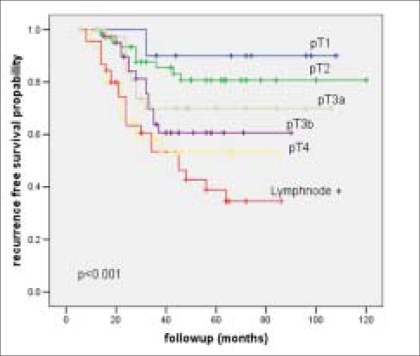
Recurrence-free survival curve stratified by pathological stage and lymph node status

**Figure 4:**
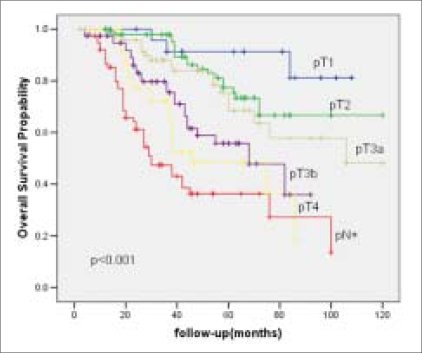
Overall survival curve stratified by pathological stage and lymph node status

The RFS and OAS were 66% and 62% at five years and it was 62% and 40% respectively at 10 years [Figures 1 and 2]. Most of the bladder cancer-related deaths occurred within three years after cystectomy. The RFS and OAS were significantly related to the pathological grade, stage and lymph node status with increasing pathological stage and lymph node positivity associated with higher rate of recurrence and worse OAS [Table 4]. The five-year RFS according to pathological stage was: pT1-92%; pT2- 80%; pT3a-72%; pT3b-60%; pT4a-40% [Figure 3] and the five-year OAS was pT1-90%; pT2-78%; pT3a-70%; pT3b-58%; pT4a-46% [Figure 4]. The five-year RFS and OAS in patients with lymph node involvement was 40% and 38% respectively.

**Table 4:** Multivariate analysis for disease-specific survival

Parameter	Relative risk	95% Cl	*P* value
Age (years)	1.132	0.821-1.912	0.724
Sex	2.074	0.961-3.112	0.621
Pathologic grade	1.65	1.021-2.320	0.010
Pathologic stage			
T1	1.000	Referent	0.042
T2	1.427	1.010-2.124	0.026
T3	2.895	1.320-3.258	0.001
T4	4.652	2.340-5.680	<0.001
Test for trend			
Lymph node metastases	3.428	1.980-4.674	<0.001
Adjuvant chemotherapy	2.435	1.210-2.986	0.684
Timing of surgery (< 3 months vs. >3 months)	1.262	1.09-1.842	0.564

### Recurrence site

A total of 145 patients (33.5%) developed bladder cancer recurrence. Of the 145 patients 40 (27.6%) developed local pelvic recurrence and 105 patients (72.4%) developed distant recurrence. Ninety per cent of recurrences occurred within 40 months after surgery. The median time to local and distant recurrence was 12 and 16 months respectively.

## DISCUSSION

Bladder cancer is the second most common genito-urinary malignancy with transitional cell carcinoma (TCC) comprising nearly 90% of primary bladder tumors. The majority of patients present with superficial bladder tumors, 20-40% either present with or develop invasive disease. Invasive TCC is usually a lethal disease requiring aggressive therapy, if not treated most of them die in two years time.[11] The aim of the treatment for any invasive bladder cancer should include: 1) Long-term survival 2) Prevention of pelvic recurrence or development of metastatic disease and 3) Better quality of life. During the past 40 years, radical cystectomy with en bloc pelvic lymphadenectomy has emerged as a gold standard for patients with high-grade muscle-invasive bladder cancer. Initially, there was lack of universal acceptance of this procedure because of high morbidity and mortality and need for urinary diversion, which was not acceptable to most of the patients and this procedure was done only in few centers. With better understanding of the pathophysiology of bladder cancer, early diagnosis, improvement in pre and prostoperative care, advances in surgical and anesthetic techniques and adjuvant chemotherapy, the outcome of RC has become better and acceptable, both to the surgeon and the patient. It has now become a common surgical procedure in the armamentarium of the urologist.

In this article, we report the outcome of a large single institution cohort of patients treated with RC+B/L PLND for bladder TCC from India. Seventeen per cent of our patients were more than 70 years old. When indicated, after adequate preoperative assessment and optimization of the patient, RC is a safe procedure in the septuagenarians and patient should not be denied surgery dependent on chronologic age.[12] Ten per cent of patients who underwent RC had presented with obstructive uropathy. Their renal function was stabilized with percutaneous nephrostomies prior to proceeding for definitive treatment. Patients of bladder cancer with obstructive uremia usually present with locally advanced disease. Our experience with these patients showed that RC is not associated with additional morbidity provided they are adequately prepared before surgery by optimizing the renal function. An adequate number of these patients achieve long-term disease-free survival after RC. Ileal conduit, as the urinary diversion appears to be safe in patients with a serum creatinine of <2.5 mg% at surgery.[13]

We found that 90% of our patients had high-grade disease and 25% had lymph node involvement. Our results showed that RC+PLND provide durable local control and long-term survival with five-year OAS and RFS rates of 62% and 66% respectively. These rates are comparable to the other reported single institution series. In a large series, Stein *et al*., reported five-year RFS and OAS of 68% and 60% respectively in patients who underwent RC and PLND over a period of 26 years.[1] The risk of recurrence increased significantly with the grade and pathological stage of the cancer. The RFS curves showed near flattening after 40 months suggesting that most of the recurrences occur within this period. Analysis of the impact of delaying the surgery did not reveal significant difference in the RFS between the two groups (those who underwent surgery within three months *vs.* >three months) [Table 4]. This could be due to the delayed primary presentation of most of the patients in either group. Patients with stage ≥ pT3b and/or lymph node involvement were considered for adjuvant chemotherapy. However, all of them did not receive chemotherapy owing to multiple factors like 1) Preexisting azotemia not improved even after urinary diversion 2) Patient denial 3) Co-morbid illnesses. On multivariate analysis, we did not observe the impact of the adjuvant chemotherapy to be significant on long-term RFS outcome.

More than 50% of our patients present at an advanced stage (extravesical disease with/without lymph node involvement). The various possible causes are (i) late presentation due to ignorance, low literacy rate and poverty (ii) A large number of patients first present to a general practitioner or a quack who try to control the disease with some medicinal means and hence, delaying the proper treatment. Ileal conduit was the predominant form of urinary diversion in our patients compared to continent diversions in the other series. The possible causes are 1) Poor compliance and follow-up due to lack of education and resources 2) Low acceptance rate for continent diversions are due to nature of job and need of intermittent self-catheterization 3) Late presentation due to ignorance, low literacy rate and poverty giving rise to high-stage disease or renal impairment. With proper selection, at least 75-90% of the patients undergoing cystectomy may be considered appropriate for ONB.[7] In a national survey it has been found that more than 50% of the institutions among the responders even today are making ileal conduit.[14] The perioperative mortality is higher compared to that reported by most of the contemporary series.[13] Systemic consequences of septic complications were the most common cause of perioperative death in our patients. Most of the septic complications are consequent to postoperative chest infection. A multidisciplinary approach with anesthesia and critical care support is important in the management of these patients. The higher incidence of septic complications in our patients could be related to a) lengthy preoperative hospital stay resulting in colonization of microbes b) inferior nutritional status of our patients with the resultant decreased resistance to infections.

The clinical results reported from this large group of patients demonstrate that radical cystectomy provides good survival results for invasive bladder cancer patients. The results from this large series of patients provide sound data and a standard with which other forms of therapy for invasive bladder cancer can be compared. It is emphasized that early detection and early referral to urologist will pick up cases for cystectomy at an early stage and will improve survival.[15]
